# Psychometric properties of the Beck Hopelessness Scale in Ecuadorian older adults: a comparative analysis of one-factor, two-factor, and three-factor models

**DOI:** 10.3389/fpsyg.2026.1911237

**Published:** 2026-09-14

**Authors:** José Alejandro Valdevila Figueira, Andrés Ramírez, Rocío Valdevila Santiesteban, Vanessa Quito-Calle, Jose A. Rodas, Pedro Andrés Muñoz-Arteaga, María José Pico Cucalon, Indira Dayana Carvajal Parra

**Affiliations:** 1 Universidad Ecotec, Samborondón, Ecuador; 2 Research Network in Psychology and Psychiatry (RIPYP), Guayaquil, Ecuador; 3 Department of Clinical Psychology, Applied Clinical Neuroscience Research Group (GINCA), Universidad Politécnica Salesiana, Cuenca, Ecuador; 4 Escuela de Psicología, Universidad Espíritu Santo, Samborondón, Ecuador; 5 School of Psychology, University College Dublin, Dublin, Ireland; 6 Casa Grande University, Guayaquil, Ecuador

**Keywords:** Beck Hopelessness Scale, older adults, psychometrics, reliability, validity

## Abstract

**Introduction:**

Hopelessness is a predictor of depression and suicide. Suicide is a preventable cause of mortality worldwide. Although it is not among the leading overall causes of death in older adults due to the predominance of chronic non-communicable diseases, it remains an epidemiologically relevant issue in this stage of life. Globally, suicide ranked as the twenty-first leading cause of death in 2021, and approximately 16.6% of suicide-related deaths occurred among individuals aged 70 years and older. This highlights the need to assess associated psychological factors, such as hopelessness and depression, in the older adult population.

**Objective:**

To evaluate the psychometric properties of the Beck Hopelessness Scale in Ecuadorian older adults by analyzing its factorial structure in one-, two-, and three-factor models, as well as its reliability and validity evidence, in order to determine the most appropriate structure for use in this population.

**Method:**

The internal factorial structure, reliability, and construct validity of the Beck Hopelessness Scale were analyzed in Ecuadorian older adults. A total of 2,233 participants were assessed. Analyses were conducted for one-, two-, and three-factor variants. Reliability was examined using McDonald’s omega coefficient and Cronbach’s alpha, while validity was assessed through confirmatory factor analysis using the Weighted Least Squares Mean and Variance Adjusted (WLSMV) estimation method.

**Results:**

The Beck Hopelessness Scale was evaluated in 2,233 Ecuadorian older adults (M = 74.02 years, SD = 8.96). Confirmatory factor analyses demonstrated an adequate fit for the one-, two-, and three-factor structures, with the one-factor model standing out due to its greater parsimony. The scale showed high internal consistency (ω = 0.933; α = 0.909) and adequate convergent validity (average variance extracted = 0.657). Factor loadings were significant across all models, supporting the relevance of the items for measuring hopelessness, although items D16 and D17 showed comparatively lower performance.

**Conclusion:**

Overall, the findings support the validity and reliability of the scale for assessing hopelessness in Ecuadorian older adults.

## Introduction

Hopelessness is a psychological construct of great relevance for understanding mental health, particularly during stages of the life cycle characterized by cumulative losses, functional changes, and increased exposure to chronic illnesses. From a cognitive perspective, hopelessness has been defined as a system of negative expectations about the future, accompanied by the perception that adverse events cannot be modified through personal action (Abramson et al., 1989; Beck et al., 1974). This disposition reflects a relatively stable way of interpreting the future through pessimism, helplessness, and the anticipation of unfavorable outcomes. In older adults, hopelessness may be associated with retirement, widowhood, functional dependency, persistent pain, social isolation, reduced family support, and the perception of a loss of meaning in life (Conwell et al., 2011; Conejero et al., 2018; Fässberg et al., 2012).

The clinical relevance of hopelessness has been supported by its association with depression, suicidal ideation, and suicidal behavior. Several studies have shown that high levels of hopelessness predict subsequent suicide among individuals with suicidal ideation and among psychiatric outpatients (Beck et al., 1985; Beck et al., 1990; Beck et al., 1993; McMillan et al., 2007; Ribeiro et al., 2018; Franklin et al., 2017).

Among the most widely used instruments for the systematic assessment of hopelessness is the Beck Hopelessness Scale (BHS), developed by Beck, Weissman, Lester, and Trexler. The scale consists of 20 dichotomous-response items designed to assess negative expectations about the future, loss of motivation, and pessimistic attitudes (Beck et al., 1974). Its use has expanded across clinical, community, university, medical, and geriatric populations for both research purposes and the identification of psychological vulnerability (McMillan et al., 2007; Ribeiro et al., 2018; Franklin et al., 2017; Hill et al., 1988; Neufeld et al., 2010; Satorres et al., 2018). However, the application of a scale within a specific population requires evidence of validity and reliability obtained in that same context, as age, culture, educational level, response patterns to positively and negatively worded items, and clinical conditions may influence the psychometric performance of the instrument (Satorres et al., 2018; Kliem et al., 2018; Hermosillo-de la Torre et al., 2020; Rueda-Jaimes et al., 2018).

The study of hopelessness in older adults is closely linked to suicide prevention. Worldwide, suicide remains a major public health concern. The Global Burden of Disease study reported that although the age-standardized suicide mortality rate decreased between 1990 and 2016, the absolute number of deaths increased due to population growth (Naghavi, 2016). More recent estimates from the Global Burden of Disease 2021 reported approximately 746,000 suicide deaths in 2021, with a greater absolute burden among men and substantial variations according to age, sex, and region (GBD 2021 Suicide Collaborators, 2025). Suicide among older adults is of particular concern because several studies have shown that rates tend to increase with age in many countries, especially among men, and that suicidal behavior in later life is often associated with higher lethality, less prior communication of suicidal intent, and a greater prevalence of physical illnesses or chronic pain (Conwell et al., 2011; Conejero et al., 2018).

In the Region of the Americas, suicide mortality has shown a concerning pattern. Unlike other regions of the World Health Organization, where rates have generally declined, the Americas have experienced increases over recent decades (Lange et al., 2023). This phenomenon has been associated with contextual factors such as social inequality, unemployment, alcohol consumption, violence, access to lethal means, and differences in the organization of mental health systems (Lange et al., 2023). This regional perspective is particularly relevant for Ecuador, where suicide rates have shown significant territorial and sociodemographic variations, particularly affecting older men (Lapo-Talledo et al., 2023).

In Ecuador, available evidence also highlights the importance of suicide as a public health issue. A geodemographic study covering the period from 2001 to 2015 reported a substantial burden of suicide mortality, with differences according to sex, province, and age group (Ortiz-Prado et al., 2017). Subsequent national studies using data from 2011 to 2020 identified more than ten thousand suicide deaths and confirmed a higher frequency among men, as well as significant territorial and sociodemographic variations (Lapo-Talledo et al., 2023). Among Ecuadorian older adults, Troya and colleagues analyzed suicides occurring between 1997 and 2019 and found considerably higher rates among men than women, underscoring the need to investigate associated psychological factors during this stage of life (Troya et al., 2021). During the COVID-19 pandemic, analyses of suicides reported by the Ecuadorian police provided insights into trends and risk factors during a period of heightened social and health vulnerability (Gerstner et al., 2022).

Differences among populations have been one of the main challenges in psychometric research on the Beck Hopelessness Scale. In psychiatric samples, hopelessness is commonly associated with depressive symptoms, suicidal ideation, and elevated clinical risk; in community samples, it may manifest with lower intensity and be more strongly influenced by social, economic, or interpersonal support variables (Beck et al., 1993; McMillan et al., 2007; Kliem et al., 2018). Among older adults, some studies have suggested that hopelessness may be related to quality of life, perceived health, self-harm risk, and future orientation (Hill et al., 1988; Neufeld et al., 2010; Satorres et al., 2018). Therefore, it is inappropriate to assume that the factor structure observed in adolescents, university students, psychiatric patients, or individuals with medical illnesses will automatically replicate in Ecuadorian older adults.

Another central issue concerns the diversity of factorial models proposed for the scale. The one-factor model conceptualizes hopelessness as a global construct and is particularly useful when the objective is to obtain a parsimonious and interpretable total score (Hermosillo-de la Torre et al., 2020; Balsamo et al., 2020). Two-factor models have been reported in studies where items appear to cluster into dimensions related to negative expectations and loss of motivation, or according to positive and negative wording effects (Pompili et al., 2007; Hanna et al., 2011). The three-factor model, in contrast, is linked to the classical tradition that distinguishes affective, motivational, and cognitive components of hopelessness. Although this model has received support in some studies, findings have not been consistent across populations (Beck et al., 1974; Dyce, 1996). Accumulated evidence suggests that differences among models may depend on sample characteristics, estimation methods, the dichotomous response format, acquiescence, and method effects associated with item wording (Kliem et al., 2018; Hermosillo-de la Torre et al., 2020; Balsamo et al., 2020).

Consequently, we considered it pertinent to evaluate the psychometric properties of the BHS in Ecuadorian older adults, given the limited evidence available for this population and the clinical importance of having reliable instruments to identify hopelessness. This study provides evidence regarding the internal structure of the instrument, its reliability, and its cultural appropriateness, while also allowing an empirical comparison of one-, two-, and three-factor models. From an applied perspective, the availability of a validated scale for Ecuadorian older adults may contribute to the early detection of emotional vulnerability, the assessment of psychological risk, and the design of preventive interventions in clinical, community, and gerontological settings.

## Materials and methods

### Study approach and design

The study was conducted under a quantitative approach, with an instrumental design, psychometric scope, and a non-experimental, cross-sectional, and analytical design. The study included a comparative psychometric component in which five competing confirmatory factor structures of the Beck Hopelessness Scale (BHS) were evaluated: a one-factor model, a correlated two-factor model, a correlated three-factor model, two-factor structure and a second-order model.

### Study population

The study population consisted of Ecuadorian older adults aged 65 years and older, residing in Ecuador and belonging to community settings in the city of Cuenca. An older adult was defined as any individual who had reached 65 years of age or older at the time of assessment. The selection of this population was based on the clinical and social relevance of hopelessness in later life, given its association with depressive symptoms, negative expectations about the future, reduced emotional well-being, suicidal ideation, and diminished quality of life.

### Sample and sampling method

The sample consisted of Ecuadorian older adults who met the established inclusion criteria and voluntarily agreed to participate in the study by providing informed consent. A non-probability convenience sampling method was employed, considering participants’ accessibility across the different data collection settings. Efforts were made to include individuals from different age groups, sexes, educational levels, and backgrounds in order to increase sample heterogeneity.

To ensure the stability of the psychometric analyses, an adequate sample size was considered for conducting confirmatory factor analysis. Sample size adequacy was determined based on the recommendation of a minimum of 10 participants per item for confirmatory factor analysis.

### Inclusion criteria

Participants were required to be 65 years of age or older, Ecuadorian nationals or permanent residents of Ecuador, capable of understanding the instrument instructions, willing to participate voluntarily in the study, and possessing sufficient cognitive and communicative abilities to complete the scale. Older adults with controlled chronic illnesses were also included, provided that these conditions did not significantly interfere with item comprehension or the response process.

### Exclusion criteria

Participants were excluded if they presented severe cognitive impairment previously diagnosed or evident during assessment, severe uncorrected sensory impairments that hindered communication, a confusional state, acute emotional crisis at the time of evaluation, active psychotic disorders, inability to provide informed consent, or refusal to continue participation.

When signs of significant emotional distress, elevated hopelessness, or potential suicide risk were identified during assessment, a protocol for psychological support, guidance, and referral to psychological or medical services was activated through the participants’ family members or caregivers, depending on the severity of the case.

### Instrument

The Beck Hopelessness Scale (BHS) (Beck et al., 1974) assesses negative expectations about the future, loss of motivation, and pessimistic attitudes toward life. The instrument consists of 20 dichotomous-response items, typically answered as true/false, from which a total hopelessness score is derived. For the present study, a validated Spanish-language version of the scale adapted for Latin American populations was used (Hermosillo-de la Torre et al., 2020). The prefix D used to label the items denotes their dichotomous response format.

Five competing confirmatory factor models were evaluated. The first was a one-factor model in which all 15 retained items loaded on a general hopelessness factor. The second was a correlated two-factor model comprising Expectations about the Future and Lack of Motivation. The third was a correlated three-factor model comprising Expectations about the Future, Lack of Motivation, and Feelings about the Future. The fourth was a two-factor structure model in which all items loaded simultaneously on a general hopelessness factor and on one corresponding specific factor; the specific factors were specified as orthogonal to the general factor and to one another. The fifth was a second-order model in which the three first-order factors loaded on a higher-order general hopelessness factor (Table 1).

**Table 1:** Confirmatory factor analysis model specifications.

Model	Factor	Indicators	N
Three-factor model	EAF	D10, D8, D4	3
	LM	D20, D17, D16, D12, D9, D3	6
	FAF	D19, D15, D13, D6, D5, D1	6
Two-factor model	EAF	D10, D8, D19, D1, D15, D13, D6, D5, D4	9
	LM	D20, D17, D16, D12, D9, D3	6
One-factor model	GHF	D10, D8, D20, D17, D16, D12, D9, D3, D19, D1, D15, D13, D6, D5, D4	15

EAF, Expectations about the Future; LM, Lack of Motivation; FAF, Feelings about the Future; GHF, General Hopelessness Factor. The original 20-item BHS was administered. Items D2, D7, D11, D14, and D18 were excluded from the confirmatory factor analysis models following preliminary item screening and exploratory factor analysis; the exclusion criteria are described in the Statistical Analysis subsection.

A sociodemographic questionnaire developed specifically for this study was administered. This questionnaire collected information on age, sex, marital status, ethnicity, educational level, and socioeconomic status.

### Procedure

The researchers visited 1,009 households in Cuenca and provided a brief explanation of the study objectives, procedures, confidentiality measures, and the voluntary nature of participation. Participants signed an informed consent form to confirm their understanding and willingness to participate. Households were selected through convenience sampling in urban sectors of Cuenca. In households with multiple eligible older adults, all consenting individuals were evaluated.

The questionnaire was administered in participants’ homes, allowing them to complete it at their convenience. No personally identifiable information, such as names, was collected in order to ensure privacy. Responses were securely stored in an encrypted database accessible only to the principal investigator, in compliance with data protection regulations.

No missing data were observed for the BHS items, thus no imputation procedures were required.

### Data analysis

Internal consistency was assessed to determine the reliability of both the individual items and the overall scale. Reliability benchmarks were interpreted as follows: ≥ 0.70 acceptable, ≥ 0.80 good, and ≥ 0.90 excellent.

The factorial structure was examined through Confirmatory Factor Analysis (CFA) using the Diagonally Weighted Least Squares (DWLS) estimator, which is appropriate for ordinal data. Model fit was evaluated using several goodness-of-fit indices, including the chi-square statistic (χ^2^), Root Mean Square Error of Approximation (RMSEA), Standardized Root Mean Square Residual (SRMR), Tucker–Lewis Index (TLI), and Comparative Fit Index (CFI). Model fit was evaluated using the following thresholds: CFI and TLI ≥ 0.95 for excellent fit, and RMSEA and SRMR ≤ 0.08 for acceptable fit (Hu and Bentler, 1999). Internal consistency was interpreted as acceptable if ≥ 0.70, and convergent validity was supported if the average variance extracted was ≥ 0.50 (Fornell and Larcker, 1981).

In addition, factor loadings and correlations among latent factors were analyzed to verify model adequacy. Items with factor loadings ≥ 0.50 were retained, indicating a substantial contribution to their respective factors. Measurement invariance of the best-supported factorial model was examined across participants who were married or living in a common-law union and participants who were widowed. A sequential multigroup confirmatory factor analysis was conducted to evaluate configural, loading, and threshold invariance for the dichotomous indicators. Measurement invariance was assessed by examining changes in model fit between increasingly constrained models. Changes of ΔCFI ≤ 0.010 and ΔRMSEA ≤ 0.015 were considered evidence that the additional equality constraints did not meaningfully worsen model fit.

Items D2, D7, D11, D14, and D18 were excluded prior to confirmatory analyses because they exhibited extreme response frequencies and exploratory factor loadings below 0.30. All analyses were conducted using R (R Core Team, 2026), with the lavaan (Rosseel et al., 2025; Rosseel, 2012), semTools (Jorgensen et al., 2026), dplyr (Wickham et al., 2026a), and readr (Wickham et al., 2026b) packages.

## Results

The analytical sample consisted of 2,233 Ecuadorian older adults, with a mean age of 74.0 years (SD = 9.0). A higher proportion of women (*n* = 1,339; 60.0%) than men (*n* = 894; 40.0%) was observed. Regarding marital status, the majority of participants were married or living in a consensual union (*n* = 1,218; 54.5%), followed by widowed individuals (*n* = 604; 27.0%). Most participants identified as Mestizo (*n* = 2,119; 94.9%) and had completed primary education (*n* = 1,062; 47.6%). The total score for the original 20-item Beck Hopelessness Scale was calculated to evaluate the severity of hopelessness in the sample. The mean BHS score was 7.69 (SD = 2.65), with scores ranging from 2 to 16. Using the conventional clinical cut-offs, the majority of the sample exhibited mild (57.5%) or moderate (40.1%) hopelessness, whereas severe hopelessness was rare (0.5%). There were no missing responses across the BHS items. The total score for the original 20-item Beck Hopelessness Scale was calculated to evaluate the severity of hopelessness in the sample. The mean BHS score was 7.69 (SD = 2.65), with scores ranging from 2 to 16. Using conventional clinical cut-offs, the majority of the sample exhibited mild or moderate hopelessness, whereas severe hopelessness was rare (Supplementary Table S5) (Table 2).

**Table 2:** Sociodemographic characteristics of the analytical sample (*N* = 2,233).

Variable	Category	n	%
Age (years)	Mean (*SD*)	75.6 (7.5)	
	Minimum–Maximum	65–103	
Sex	Male	894	40.0
	Female	1,339	60.0
Marital status	Single	171	7.7
	Married/common-law union	1,218	54.5
	Separated/divorced	240	10.7
	Widowed	604	27.0
Ethnicity	White	35	1.6
	Mestizo	2,119	94.9
	Indigenous	75	3.4
	Afro-Ecuadorian/Afro-descendant	4	0.2
Education level	Primary education	1,062	47.6
	Secondary education	628	28.1
	Technical or higher education	215	9.6
	Postgraduate	10	0.4
	No formal education	318	14.2
Economic income in previous month	Yes	1,695	75.9
	No	538	24.1

Income encompasses financial support from family, pensions, formal or informal employment, rental or banking income, and government subsidies. A ‘No’ response indicates a complete absence of these sources.

Confirmatory factor analyses were conducted to evaluate the factorial structure. The global fit indices (Table 3) showed that the one-, two-, three-, and second-order models demonstrated adequate and highly similar psychometric performance. The three-factor and second-order models yielded identical fit indices (CFI = 0.989, TLI = 0.987, RMSEA = 0.078, SRMR = 0.074). The two-factor model showed comparable values (RMSEA = 0.077), whereas the one-factor model presented an RMSEA of 0.076. These findings indicated that all structures adequately reproduced the matrix of relationships among the items; however, the one-factor model exhibited a slight advantage in terms of parsimony. A bifactor model was also evaluated but yielded an inadmissible solution characterised by negative residual variances and a non-positive definite latent covariance matrix; therefore, its fit indices were not interpreted.

**Table 3:** Global fit comparison of the CFA models.

Model	CFI	TLI	RMSEA [90% CI]	SRMR
One-factor model	0.989	0.987	0.076 [0.073, 0.080]	0.074
Two-factor model	0.989	0.987	0.077 [0.073, 0.081]	0.074
Three-factor model	0.989	0.987	0.078 [0.074, 0.082]	0.074
Second-order model	0.989	0.987	0.078 [0.074, 0.082]	0.074

CFI, comparative fit index; TLI, Tucker–Lewis index; RMSEA, root mean square error of approximation; CI, confidence interval; SRMR, standardised root mean square residual.

Standardised factor loadings were statistically significant across all models, indicating that the indicators contributed meaningfully to their respective factors. In the three-factor model, factor loadings ranged from 0.667 to 0.888 for EAF, from 0.408 to 0.939 for LM, and from 0.804 to 0.909 for FAF. Items D12, D20, D15, D13, D19, and D6 exhibited the highest loadings and were therefore identified as the most representative indicators of the construct (Figure 1). In the one-factor model, factor loadings ranged from 0.406 to 0.935, demonstrating that most items were strongly associated with the general hopelessness factor. However, items D16 and D17 showed the lowest factor loadings and the smallest proportions of explained variance, suggesting that these indicators contributed less strongly to the latent construct. This attenuated performance may stem from linguistic nuances in the Ecuadorian context, where the phrasing of these negatively worded items might not fully capture the intended cognitive dimension of hopelessness among older adults.

**Figure 1:**
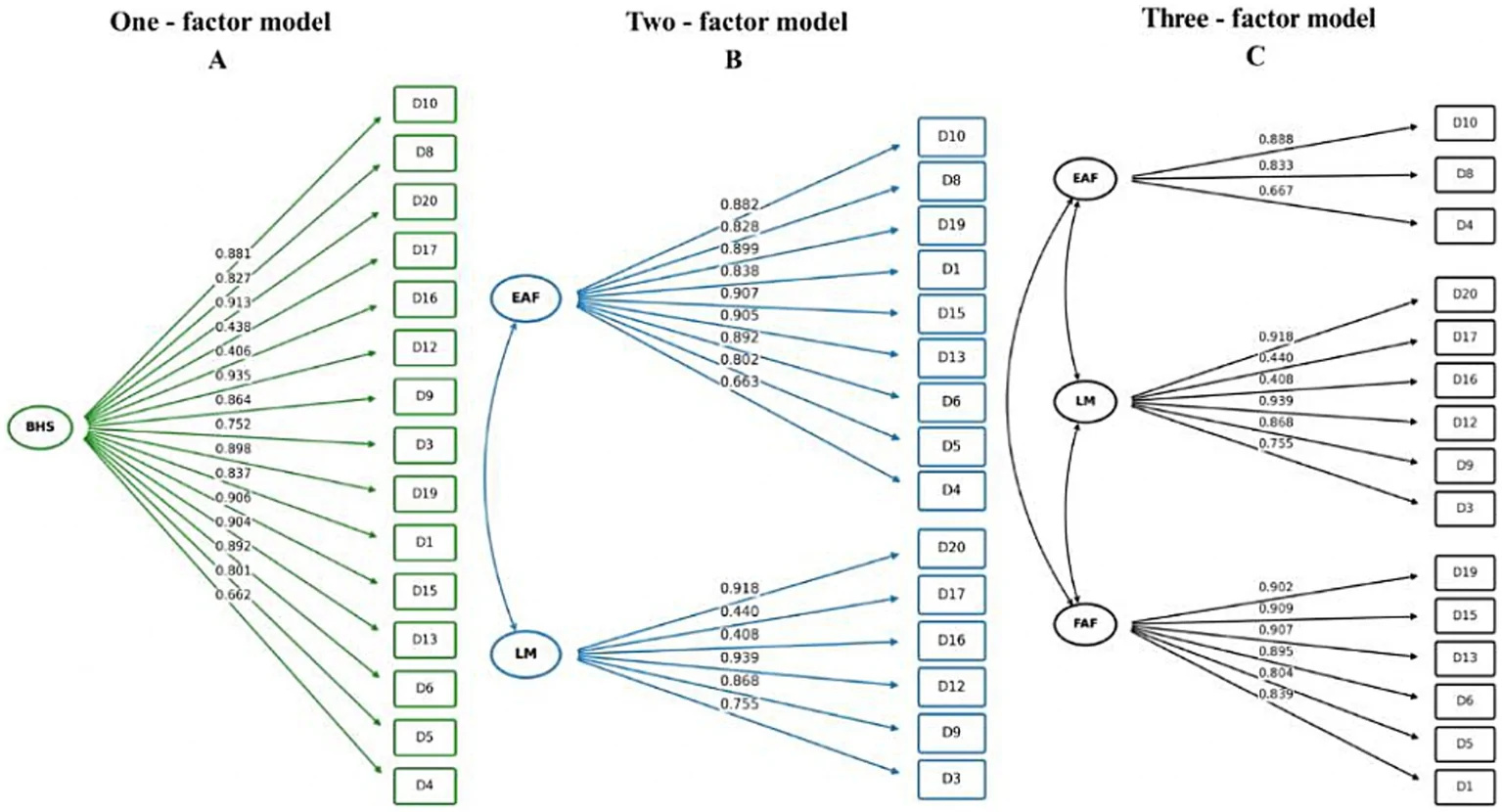
Confirmatory factor analysis models of the Beck Hopelessness Scale (BHS). Panel **A** presents the one-factor model, Panel **B** presents the two-factor model, and Panel **C** presents the three-factor model. Standardized factor loadings are shown along the corresponding paths. EAF, Expectations about the Future; LM, Lack of Motivation; FAF, Feelings about the Future.

Internal consistency was adequate across the evaluated models (Table 4). In the three-factor model, the FAF dimension showed the highest reliability coefficients (ω = 0.879, α = 0.870), whereas LM demonstrated acceptable reliability (ω = 0.746, α = 0.742). The EAF dimension exhibited low-to-moderate internal consistency (ω = 0.678, α = 0.652). In both the two-factor and one-factor models, overall reliability was high (ω = 0.933, α = 0.909), supporting the use of a total hopelessness score. Sensitivity analyses for the one-factor model confirmed that internal consistency remained stable across sex (Males: ω = 0.922, α = 0.914; Females: ω = 0.914, α = 0.906) and age groups (65–74 years: ω = 0.925, α = 0.917; ≥ 75 years: ω = 0.907, α = 0.900).

**Table 4:** Internal consistency and convergent validity by CFA model.

**Model**	**Factor**	**ω**	**α**	**AVE**
One-factor model	GHF	0.932	0.909	0.657
Two-factor model	EAF	0.898	0.891	0.722
	LM	0.746	0.742	0.568
	Total	0.933	0.909	
Three-factor model	EAF	0.678	0.652	0.642
	LM	0.746	0.742	0.568
	FAF	0.879	0.870	0.769
	Total	0.933	0.909	

ω, McDonald’s omega; α, Cronbach’s alpha; AVE, average variance extracted. AVE is not applicable to total-score reliability.

Convergent validity was supported by the average variance extracted (AVE) values. In the three-factor model, FAF achieved an AVE of 0.769, EAF obtained 0.642, and LM showed 0.568. In the two-factor model, the broader factor representing expectations and feelings about the future yielded an AVE of 0.722, whereas the one-factor model presented an AVE of 0.657. These results indicate that the factors explained an adequate proportion of the variance in their respective indicators.

Measurement invariance of the one-factor model was examined across participants who were married or living in a common-law union (*n* = 1,218) and those who were widowed (n = 604). The configural model showed adequate fit across the two groups. Imposing equality constraints on factor loadings and item thresholds did not result in a meaningful deterioration in model fit (Δ_CFI_ = 0.016, Δ_RMSEA_ = −0.025). Therefore, full scalar invariance was supported, indicating that the items function similarly regardless of marital status.

Following the establishment of scalar invariance, a latent mean comparison was conducted. The latent mean of the married group was fixed to zero to serve as the reference category. The latent mean of the widowed group was −0.22 (SE = 0.046, *p* < 0.001), with a standardised difference of −0.25. This indicates significantly lower levels of hopelessness among widowed participants compared to those who are married or in a common-law union.

## Discussion

The present study comparatively examined the one-, two-, and three-factor structures of the Beck Hopelessness Scale (BHS) in a sample of 2,233 Ecuadorian older adults. Overall, the findings support the psychometric adequacy of the scale in this population and provide relevant evidence for its use in gerontological research, community mental health, and psychosocial assessment.

The sample, with a mean age of 74.02 years, a predominance of women, a majority of participants who were married or living in a consensual union, predominantly Mestizo ethnic identification, and mainly primary-level education, represents a sociodemographically relevant context for analyzing the functioning of the BHS in older adults. Hopelessness in later life may be influenced by educational, economic, familial, cultural, and health-related conditions that shape how individuals perceive their future.

The global fit indices showed that all three evaluated models demonstrated adequate and highly similar performance, suggesting that hopelessness can be understood both as a general construct and as an experience composed of affective, motivational, and cognitive components. This interpretation is consistent with the original proposal of Beck et al. (1974), who conceptualized hopelessness as a set of negative expectations about the future expressed through affective, motivational, and cognitive dimensions. Likewise, subsequent clinical studies have identified multidimensional structures, such as the three-factor solution described by Dyce in outpatient populations (Dyce, 1996) and the three-trait, two-method-factor model proposed Boduszek and Dhingra (2016).

However, although the three-dimensional model retains theoretical value, the results of the present study favor the use of the one-factor model because of its parsimony, reliability, and interpretability. The similarity of the fit indices indicates that none of the evaluated structures was clearly superior from a statistical perspective. Nevertheless, when competing models show equivalent performance, the simpler solution is generally preferable, particularly when it allows for the calculation of a stable and clinically meaningful total score. This conclusion is consistent with previous studies supporting one-factor model solutions for the BHS. Kliem et al. (2018) in a representative German sample, found a one-factor model structure to be adequate and highlighted its usefulness for calculating and interpreting a total score. Similarly, Aish et al. (2001) reported that two- and three-factor models did not fit satisfactorily among individuals with a history of suicide attempts, whereas simpler structures adequately explained the relationships among items. Hanna et al. (2011) in a non-clinical sample from the United Kingdom, also concluded that the BHS could be represented by a brief one-factor model model.

Recent evidence based on Item Response Theory has also supported the trend toward more parsimonious solutions. Balsamo et al. identified a nine-item version with one-factor model properties and adequate discriminative capacity for suicide risk among psychiatric patients (Balsamo et al., 2020). Pretorius and Padmanabhanunni (2024) proposed a seven-item short form that retained internal consistency and criterion validity in a non-clinical population. Likewise, Bottaro and Faraci (2024) developed a ten-item brief version that adequately represented the trait continuum measured by the original scale and showed associations with suicidal orientation. In this context, the present findings align with a contemporary trend in the psychometric literature: although the BHS may retain a multidimensional conceptual basis, its empirical application often benefits from the use of a total hopelessness score.

The results of the present study are also consistent with Latin American research that has confirmed the psychometric usefulness of the BHS in culturally and socially specific populations. In Mexico, Quiñonez Tapia et al. (2019) evaluated the scale in 771 high school and university students living in conditions of poverty and reported adequate internal consistency using McDonald’s omega, as well as satisfactory fit indices in confirmatory factor analysis. Although that study was not conducted with older adults, its contribution is relevant because it demonstrates that the BHS can function adequately in Latin American populations exposed to socioeconomic vulnerability, where hopelessness is particularly important because of its association with depression and suicide. Furthermore, it reinforces the need to validate the instrument’s internal structure and reliability within specific groups, given that social, economic, and cultural conditions may influence the expression of the construct.

Similarly, Pineda-Roa et al. (2024) reported an adequate one-factor model structure, high internal consistency, and nomological validity with depressive symptoms and suicide risk among Colombian adolescents. Likewise, Flores-Kanter et al. (2022) emphasised the importance of considering method effects associated with positively and negatively worded items, proposing a correlated traits-correlated (method-1) model to more accurately distinguish the hopelessness trait from method-related variance. Collectively, these Latin American studies help contextualise the Ecuadorian findings and suggest that the BHS is a promising instrument for measuring hopelessness in the region, although its factorial structure may vary according to age, educational level, socioeconomic context, sample characteristics, and linguistic factors.

## Limitations

The high proportion of Mestizo participants limits the generalizability of the findings to Indigenous, Afro-Ecuadorian, and other ethnic groups within Ecuador. Furthermore, although variables such as age, sex, educational level, ethnicity, marital status, and income during the previous month were collected, the results presented do not include analyses of factorial invariance or differential item functioning across these subgroups. This represents a priority area for future research, particularly because educational level, income, and marital status may influence both the comprehension of questionnaire items and the subjective experience of hopelessness.

Additionally, it would be advisable to examine the convergent and discriminant validity of the Beck Hopelessness Scale (BHS) in relation to depressive symptoms, loneliness, social support, functional status, quality of life, resilience, and suicidal ideation among Ecuadorian older adults.

## Data Availability

The raw data supporting the conclusions of this article will be made available by the authors, without undue reservation.
