# Supplementary material for: Psychometric properties of the Beck Hopelessness Scale in Ecuadorian older adults: a comparative analysis of one-factor, two-factor, and three-factor models
Source: Front Psychol. 2026 Sep 14;17:1911237. doi: 10.3389/fpsyg.2026.1911237 (PMC13616808; doi:10.3389/fpsyg.2026.1911237)
Supplement: Supplementary file 1 [file Table_1.DOCX]

**Supplementary Tables**

*Psychometric Properties of the Beck Hopelessness Scale in Ecuadorian Older Adults: A Comparative Analysis of Unifactorial, Bifactorial, and Trifactorial Models*

**Supplementary Table S1. Item-level descriptive statistics for the Beck Hopelessness Scale (D1-D20)**

| **Item** | **Spanish item wording used in the dataset** | **0 = No, n (%)** | **1 = Yes, n (%)** |
| --- | --- | --- | --- |
| D1 | Espera el futuro con esperanza y entusiasmo | 526 (23.6%) | 1707 (76.4%) |
| D2 | Se ha dado por vencido, ha renunciado, ya que no ha podido hacer mejor las cosas por sí mismo | 1410 (63.1%) | 823 (36.9%) |
| D3 | Cuando las cosas van mal le alivia saber que las cosas no pueden permanecer todo el tiempo así | 614 (27.5%) | 1619 (72.5%) |
| D4 | Ha podido imaginar cómo será su vida dentro de 10 años | 1012 (45.3%) | 1221 (54.7%) |
| D5 | Tiene bastante tiempo para llevar a cabo las cosas que quisiera poder hacer | 732 (32.8%) | 1501 (67.2%) |
| D6 | En el futuro, espera conseguir lo que le pueda interesar | 639 (28.6%) | 1594 (71.4%) |
| D7 | Su futuro le parece oscuro | 1714 (76.8%) | 519 (23.2%) |
| D8 | Espera más cosas buenas de la vida de lo que la gente suele conseguir por término medio | 727 (32.6%) | 1506 (67.4%) |
| D9 | Ha logrado hacer que las cosas cambien, y existen razones para creer que pueda hacerlo en el futuro | 614 (27.5%) | 1619 (72.5%) |
| D10 | Sus experiencias pasadas le han preparado bien para su futuro | 451 (20.2%) | 1782 (79.8%) |
| D11 | Todo lo que puede ver por delante de usted es más desagradable que agradable | 1526 (68.3%) | 707 (31.7%) |
| D12 | Espera conseguir lo que realmente desea | 519 (23.2%) | 1714 (76.8%) |
| D13 | Cuando mira hacia el futuro, espera que sea más feliz de lo que es ahora | 532 (23.8%) | 1701 (76.2%) |
| D14 | Las cosas no marchan como usted quisiera | 1381 (61.8%) | 852 (38.2%) |
| D15 | Tiene una gran confianza en su futuro | 629 (28.2%) | 1604 (71.8%) |
| D16 | Nunca ha conseguido lo que desea, por lo que es absurdo desear cualquier cosa | 1413 (63.3%) | 820 (36.7%) |
| D17 | Es muy improbable que pueda lograr una satisfacción real en el futuro | 1230 (55.1%) | 1003 (44.9%) |
| D18 | El futuro le parece vago e incierto | 1415 (63.4%) | 818 (36.6%) |
| D19 | Espera más bien épocas buenas que malas | 512 (22.9%) | 1721 (77.1%) |
| D20 | Merece la pena que intente conseguir algo que desee, porque probablemente lo logre | 522 (23.4%) | 1711 (76.6%) |

***Note.*** *N = 2,233. Percentages are calculated over the complete analytic sample. (0 = No; 1 = Yes).*

**Supplementary Table S2. Score ranges and cutoff status for the standard BHS and the factor models evaluated in the manuscript**

| **Scoring structure** | **Factor / score** | **Items** | **k** | **Possible range** | **Hopelessness severity cutoff / status** |
| --- | --- | --- | --- | --- | --- |
| Standard BHS-20 | Total score | D1-D20 | 20 | 0-20 | Conventional bands: 0-3 minimal; 4-8 mild; 9-14 moderate; 15-20 severe. |
| One-factor model | GHF | D10, D8, D20, D17, D16, D12, D9, D3, D19, D1, D15, D13, D6, D5, D4 | 15 | 0-15 | No validated model-specific clinical cutoffs established. |
| Two-factor model | EAF | D10, D8, D19, D1, D15, D13, D6, D5, D4 | 9 | 0-9 | No validated factor-specific clinical cutoffs established. |
| Two-factor model | LM | D20, D17, D16, D12, D9, D3 | 6 | 0-6 | No validated factor-specific clinical cutoffs established. |
| Three-factor model | EAF | D10, D8, D4 | 3 | 0-3 | No validated factor-specific clinical cutoffs established. |
| Three-factor model | LM | D20, D17, D16, D12, D9, D3 | 6 | 0-6 | No validated factor-specific clinical cutoffs established. |
| Three-factor model | FAF | D19, D15, D13, D6, D5, D1 | 6 | 0-6 | No validated factor-specific clinical cutoffs established. |

***Note.*** *The conventional 0-3/4-8/9-14/15-20 severity bands apply to the standard 20-item BHS total score under the standard scoring key. Published evidence describes reverse scoring for items D1, D3, D5, D6, D8, D10, D13, D15, and D19 before summing the 20-item total. The model-specific columns in the supplied spreadsheet are raw 0/1 sums and should therefore not be interpreted with the conventional clinical severity bands. For the manuscript, GHF, EAF, LM, and FAF scores are best treated as continuous model/factor scores unless separate cutoff validation is undertaken. GHF = General Hopelessness Factor; EAF = Expectations about the Future; LM = Lack of Motivation; FAF = Feelings about the Future.*

**Scoring references:** Kliem S, Lohmann A, Mößle T, Brähler E. Psychometric properties and measurement invariance of the Beck Hopelessness Scale: results from a German representative population sample. BMC Psychiatry. 2018;18. Balsamo M, Carlucci L, Innamorati M, Lester D, Pompili M. Further insights into the Beck Hopelessness Scale: unidimensionality among psychiatric inpatients. Front Psychiatry. 2020;11.

**Supplementary Table S3. Item-level descriptive statistics for the Beck Hopelessness Scale by sex**

| **Item** | **Spanish item wording used in the dataset** | **Men (n = 894)** | | **Women (n = 1,339)** | |
| --- | --- | --- | --- | --- | --- |
|  |  | **0 = No, n (%)** | **1 = Yes, n (%)** | **0 = No, n (%)** | **1 = Yes, n (%)** |
| D1 | a. Espera el futuro con esperanza y entusiasmo | 192 (21.5%) | 702 (78.5%) | 334 (24.9%) | 1005 (75.1%) |
| D2 | b. Se ha dado por vencido, ha renunciado, ya que no ha podido hacer mejor las cosas por sí mismo | 547 (61.2%) | 347 (38.8%) | 863 (64.5%) | 476 (35.5%) |
| D3 | c. Cuando las cosas van mal le alivia saber que las cosas no pueden permanecer todo el tiempo así | 247 (27.6%) | 647 (72.4%) | 367 (27.4%) | 972 (72.6%) |
| D4 | d. Ha podido imaginar cómo será su vida dentro de 10 años | 383 (42.8%) | 511 (57.2%) | 629 (47.0%) | 710 (53.0%) |
| D5 | e. Tiene bastante tiempo para llevar a cabo las cosas que quisiera poder hacer | 278 (31.1%) | 616 (68.9%) | 454 (33.9%) | 885 (66.1%) |
| D6 | f. En el futuro, espera conseguir lo que le pueda interesar | 234 (26.2%) | 660 (73.8%) | 405 (30.2%) | 934 (69.8%) |
| D7 | g. Su futuro le parece oscuro | 688 (77.0%) | 206 (23.0%) | 1026 (76.6%) | 313 (23.4%) |
| D8 | h. Espera más cosas buenas de la vida de lo que la gente suele conseguir por término medio | 282 (31.5%) | 612 (68.5%) | 445 (33.2%) | 894 (66.8%) |
| D9 | i. Ha logrado hacer que las cosas cambien, y existen razones para creer que pueda hacerlo en el futuro | 233 (26.1%) | 661 (73.9%) | 381 (28.5%) | 958 (71.5%) |
| D10 | j. Sus experiencias pasadas le han preparado bien para su futuro | 177 (19.8%) | 717 (80.2%) | 274 (20.5%) | 1065 (79.5%) |
| D11 | k. Todo lo que puede ver por delante de usted es más desagradable que agradable | 609 (68.1%) | 285 (31.9%) | 917 (68.5%) | 422 (31.5%) |
| D12 | l. Espera conseguir lo que realmente desea | 194 (21.7%) | 700 (78.3%) | 325 (24.3%) | 1014 (75.7%) |
| D13 | m. Cuando mira hacia el futuro, espera que sea más feliz de lo que es ahora | 195 (21.8%) | 699 (78.2%) | 337 (25.2%) | 1002 (74.8%) |
| D14 | n. Las cosas no marchan como usted quisiera | 539 (60.3%) | 355 (39.7%) | 842 (62.9%) | 497 (37.1%) |
| D15 | o. Tiene una gran confianza en su futuro | 238 (26.6%) | 656 (73.4%) | 391 (29.2%) | 948 (70.8%) |
| D16 | p. Nunca ha conseguido lo que desea, por lo que es absurdo desear cualquier cosa | 569 (63.6%) | 325 (36.4%) | 844 (63.0%) | 495 (37.0%) |
| D17 | q. Es muy improbable que pueda lograr una satisfacción real en el futuro | 492 (55.0%) | 402 (45.0%) | 738 (55.1%) | 601 (44.9%) |
| D18 | r. El futuro le parece vago e incierto | 581 (65.0%) | 313 (35.0%) | 834 (62.3%) | 505 (37.7%) |
| D19 | s. Espera más bien épocas buenas que malas | 204 (22.8%) | 690 (77.2%) | 308 (23.0%) | 1031 (77.0%) |
| D20 | t. Merece la pena que intente conseguir algo que desee, porque probablemente lo logre | 206 (23.0%) | 688 (77.0%) | 316 (23.6%) | 1023 (76.4%) |

***Note.*** *Percentages are calculated within sex. Sex coding in the dataset is 1 = man and 2 = woman. No inferential comparison is implied by this descriptive table.*

**Supplementary Table S4. Sensitivity analysis of internal consistency across sex and age strata for the one-, two-, and three-factor models**

| **Model** | **Factor** | **k** | **Total α** | **Men α (n=894)** | **Women α (n=1,339)** | **Age 65-74 α (n=1,010)** | **Age ≥75 α (n=1,008)** | **Max \|Δα\|** | **Sensitivity interpretation** |
| --- | --- | --- | --- | --- | --- | --- | --- | --- | --- |
| One-factor model | GHF | 15 | 0.909 | 0.914 | 0.906 | 0.921 | 0.900 | 0.012 | Good and stable |
| Two-factor model | EAF | 9 | 0.891 | 0.896 | 0.887 | 0.906 | 0.873 | 0.017 | Good and stable |
| Two-factor model | LM | 6 | 0.742 | 0.756 | 0.733 | 0.777 | 0.720 | 0.034 | Acceptable and stable |
| Three-factor model | EAF | 3 | 0.652 | 0.675 | 0.637 | 0.702 | 0.603 | 0.049 | Below .70; weakest in older subgroup |
| Three-factor model | LM | 6 | 0.742 | 0.756 | 0.733 | 0.777 | 0.720 | 0.034 | Acceptable and stable |
| Three-factor model | FAF | 6 | 0.870 | 0.873 | 0.868 | 0.891 | 0.847 | 0.023 | Good and stable |

***Note.*** *Cronbach's alpha was recalculated using the same raw 0/1 item coding and model-specific item sets used in the manuscript, allowing direct comparison with the reported full-sample α coefficients. Age-stratified sensitivity analyses were restricted to participants aged 65 years or older: 215 records with age <65 in the supplied database were excluded from the age-stratified calculations because they are inconsistent with the manuscript's stated inclusion criterion. Interpretive benchmarks follow those specified in the manuscript (≥ .70 acceptable, ≥ .80 good, ≥ .90 excellent). This table is a sensitivity analysis of internal consistency, not a formal test of measurement invariance. Claims of equivalent measurement properties across sex or age require multigroup CFA for categorical indicators (e.g., configural and progressively constrained invariance models).*

**Sensitivity summary.** Internal consistency was highly stable for the one-factor GHF and generally stable for the two-factor EAF and LM and the three-factor FAF and LM across sex and age strata. The three-factor EAF remained the weakest component: α = .652 in the total sample, .675 in men, .637 in women, .702 among participants aged 65-74 years, and .603 among those aged ≥75 years. Accordingly, the three-factor EAF should be interpreted cautiously, particularly in the older age subgroup.

**Supplementary Table S5.** Distribution of Hopelessness Severity Based on BHS Cut-offs (N = 2,233)

| **Severity Range** | **n** | **%** |
| --- | --- | --- |
| Minimal (0–3) | 43 | 1.9 |
| Mild (4–8) | 1,283 | 57.5 |
| Moderate (9–14) | 896 | 40.1 |
| Severe (15–20) | 11 | 0.5 |
